# Quality of Life With a Hernia—A Novel Patient Led Study

**DOI:** 10.3389/jaws.2023.11214

**Published:** 2023-04-28

**Authors:** Susannah Hill, Jackie Bullock, David Lars Sanders

**Affiliations:** ^1^ British Hernia Society Patient Representative, Manchester, United Kingdom; ^2^ North Devon District Hospital, Barnstaple, United Kingdom

**Keywords:** incisional hernia, quality of life, hernia, abdominal wall, parastomal hernia

## Abstract

**Introduction:** Abdominal wall hernia surgery aims to relieve symptoms and to improve quality of life (QoL). The aim of this novel patient led research, was to help surgeons understand how hernias impact on patients’ wellbeing.

**Methods:** A questionnaire was developed by patient advocates. It was promoted through social media to gather anonymous feedback from patients.

**Results:** 264 questionnaires were completed. The majority of the respondents were female (78.4%, *n* = 207), from English speaking countries (85.2%, *n* = 225), and had either parastomal hernias (36.0%, *n* = 95) or incisional hernias (28.0%, *n* = 74). Respondents described how their hernia affected sexual intimacy, either due to the dislike of their physical appearance, pain or the practicalities of their hernia getting in the way. They reported that their hernia restricted them from engaging in certain exercise activities, and a significant proportion also reported an impact on their diet.

**Conclusion:** This study has identified that in addition to functional problems, living with a hernia can affect mental health as well as social and physical relationships. Existing hernia QoL tools are limited in the outcomes that they measure. Without a comprehensive hernia specific QoL tool, it remains difficult for a surgeon to accurately assess the impact that different treatment modalities may have on patients.

## Introduction

Abdominal wall hernia surgery aims to relieve symptoms and to improve quality of life. Being predominantly elective surgery, this allows for preoperative time to plan and focus on patient priorities. Through understanding how a hernia affects the patient’s QoL, the surgeon can assess the impact that any potential surgery may have on them. Although hernia specific QoL tools exist, none are universally accepted (1), and most have been developed without patient input (2). Within hernia research, generic QoL tools are often used, however as not disease specific these may not capture key aspects important to patients with hernias (1).

This patient led study aims to form a greater understanding of how abdominal wall hernias impact on patients’ wellbeing, and to evaluate whether existing QoL tools ask the questions important to patients.

## Methods

The patient representatives of the British Hernia Society (BHS) were invited to present at the European Hernia Society (EHS) conference 2022, on the patient’s perspective of living with a hernia. The representatives chose to specifically focus on topics not traditionally discussed in QoL surveys, including sex, alcohol consumption, diet, and exercise.

In preparation for the conference, a 25-question patient questionnaire was developed by the patient authors using Google Forms. The authors invited three patients with different hernia types (incisional, inguinal and hiatus) to test the questionnaire and provide feedback on ease of completion, clarity of the questions, whether the questionnaire was suitable for their hernia type, and if they wanted any other questions included. Based on their feedback, the questionnaire was refined. A copy of the survey is shown in Supplementary Appendix SA1.

The questionnaire was publicised on social media. Tweets were made on Twitter using the personal profiles of the authors, these posts were retweeted by other patients and surgeons. Posts promoting the questionnaire were also made within Facebook patient support groups relating to abdominal wall hernias or stomas. The groups were selected based on their number of members. Support groups relating to the use of surgical mesh were excluded, to ensure that the results focused on living with a hernia, and not the outcomes of surgery. Some support group administrators did not respond to the request for the link to be posted within their group, none directly declined.

As this research was patient led, without the support of an institution or organisation, no ethical approval could be sought from a research ethics committee. Patients were invited to voluntarily complete the questionnaire; they were informed that their anonymised answers would be presented to surgeons, to help them gain a greater understanding on how hernias can affect a patient’s physical and emotional wellbeing. Implied consent was gained when the patients completed the questionnaire.

At the beginning of the questionnaire, patients were warned that some questions may be emotionally difficult, and at the end there were links to support groups for alcohol or substance use.

## Data Collection and Analysis

The survey was promoted over a 6-week period in March and April 2022. Participants completed the survey using an anonymous link, with the option to provide an email address to receive a summary of the results. Respondents who have already had hernia surgery, were asked to complete the questionnaire based on their experiences prior to this surgery.

The results were exported from Google Forms, analysed in Microsoft Excel by SH, and shared with JB for verification. Free text quotes were selected through manual thematic analysis, to ensure all themes were represented, across all hernia types.

DS was later invited to proof read the article and provide guidance on the submission process for publication. DS offered advice only, with all decision making and analysis performed by the patients themselves.

## Results

### Demographics

In total, 264 patients completed the survey. The majority were female (78%, *n* = 207), from English speaking countries (85%, *n* = 225), and had either parastomal hernias (36%, *n* = 95) or incisional hernias (28%, *n* = 74). Age, gender, country/region of residence and type of hernia are shown in Table 1.

**TABLE 1:** Baseline demographics, and surgical data.

Age	%	N
18–29	5.3	(14)
30–39	12.5	(33)
40–49	26.1	(69)
50–59	23.9	(63)
60–69	21.2	(56)
70 or older	10.6	(28)
Prefer not to say	0.4	(1)

Gender	%	n
Female	78.4	(207)
Male	20.8	(55)
Other	0.8	(2)

Country/Region	%	n
United Kingdom	48.9	(129)
Other European country	2.3	(6)
Africa	1.9	(5)
Asia	3.8	(10)
North America	36.4	(96)
Oceania & Australia	3.4	(9)
South America	0.8	(2)
Prefer not to say	2.7	(7)

Hernia type	%	n
Parastomal	36.0	(95)
Incisional	28.0	(74)
Umbilical	14.8	(39)
Inguinal or femoral	11.0	(29)
Epigastric	4.2	(11)
Other	6.1	(16)

**FIGURE 1:**
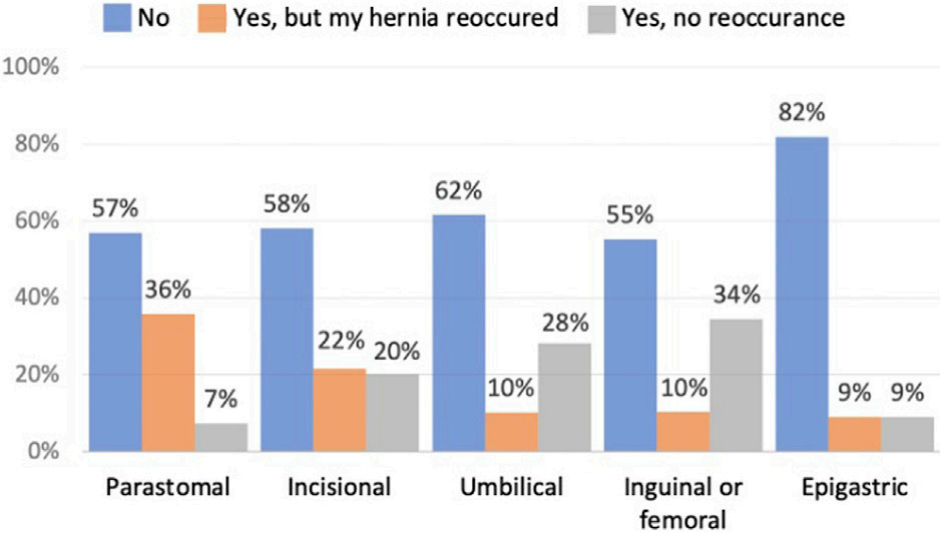
Whether patients have had hernia repair surgery.

Forty-five of the respondents (17%) reported that they no longer had a hernia, and sixty (23%) had reoccurrence after prior hernia surgery. Reoccurrence rates by hernia type are shown in Figure 1.

### Sex and Intimacy

**FIGURE 2:**
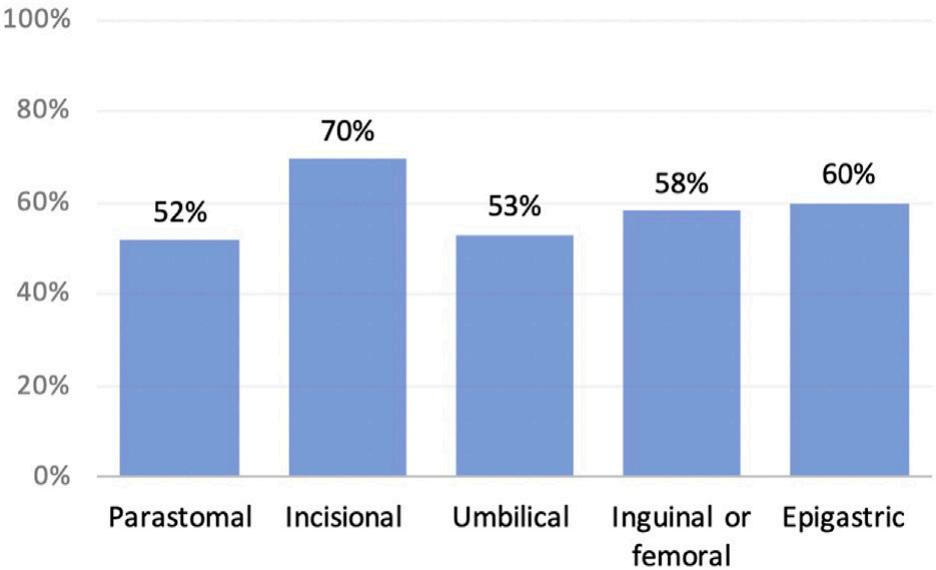
Negative impact on sex by hernia type.

**FIGURE 3:**
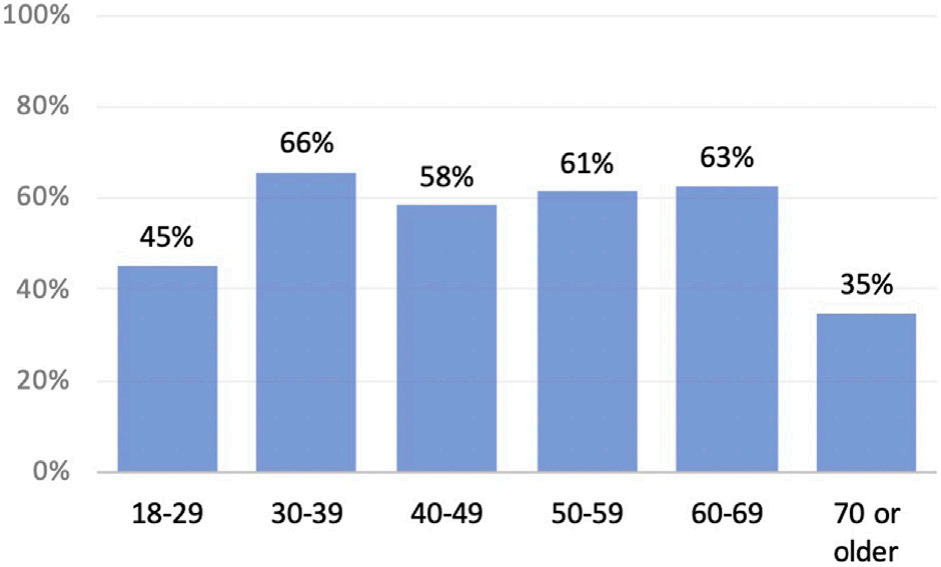
Negative impact on sex by age.

When asked about sex, fifty eight percent of respondents (*n* = 134) said that their hernia had a negative impact. The impact was across all types of hernias and age groups, as shown in Figures 2, 3.

There were three common themes within the free text: physical appearance (43%, *n* = 58), pain (31%, *n* = 42) and the practicalities of the hernia getting in the way (22%, *n* = 29). Respondents commonly used negative descriptive words such as “ugly,” “gross” and “deformed” to describe their bodies, and expressions of how they did not want their partners to see them naked. Some respondents wrote how they avoided intimacy due to discomfort or adapted to use sexual positions where their pain was less. Others needed to experiment with different sexual positions for practical reasons, to avoid the hernia getting squashed, particularly if the hernia was not reducible.

Free text: Please explain why your hernia affects your sex life

• “Positions are difficult, confidence is difficult. Pain during makes me anxious and panicky.”—Incisional hernia
• “Has not affected negatively, just had to adapt”—Inguinal/femoral hernia
• “It is gross and I feel disgusting, so I do not want anybody to touch me, much less look at me”—Parastomal hernia
• “It gets smashed in the process”—Umbilical hernia
• “I feel deformed and unattractive, and my partner is afraid to ‘break’ me any further”—Incisional hernia -

### Exercise

**FIGURE 4:**
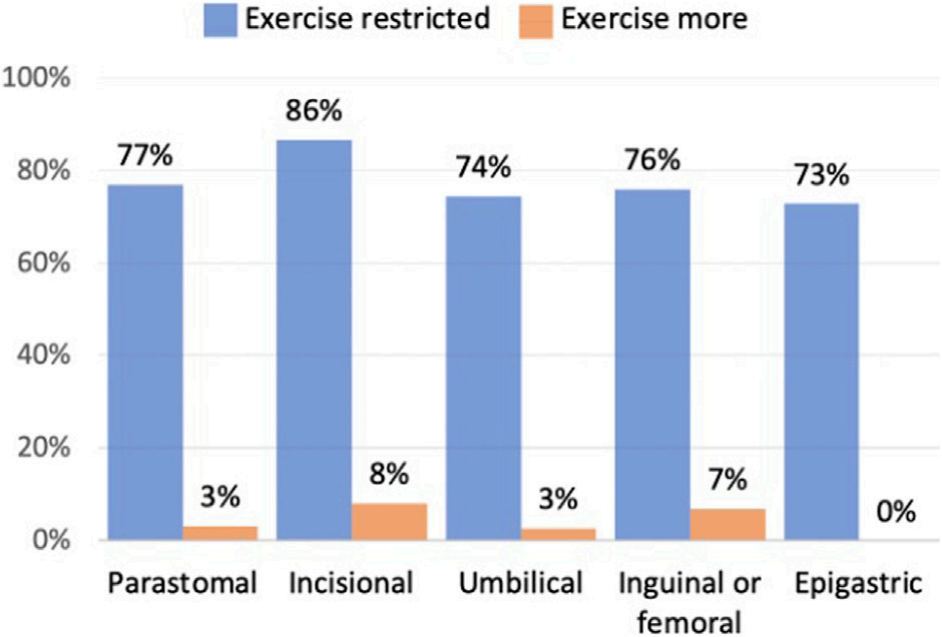
Does your hernia affect your excercise routine.

The majority of respondents (79%, *n* = 209) said that they are limited on the exercise that they could do because of their hernia, this was across all hernia types as shown in Figure 4. Of these, forty seven percent (*n* = 98) said they had been instructed to avoid certain activities. It is not known whether those restrictions were advised by their surgeon, other healthcare professionals or other patients.

Other main reasons respondents gave for not being able to do certain exercises were pain and discomfort (31%, *n* = 65) and avoiding using or reduced mobility through their core (24%, *n* = 51). Some respondents described fear of making their hernia worse (10%, *n* = 21), whilst others said that their hernia got in the way (4%, *n* = 8). Within the free text there was an underlying sense of frustration, from respondents not being able to do the activities that they used to do or wanted to do, including group activities and social sports such as golf, sailing or basketball.

Free text: If your hernia affects your exercise routine, please explain how and why

• “I am scared to make it worse, so walking is the only exercise I feel relatively safe doing. It inhibits me from doing things like gardening let alone sports like tennis and skiing that I used to love.”—Incisional hernia
• “Used to love going long walks with my dog, but now it is shorter walks so less exercise” - Parastomal hernia
• “I feel I can not play sports like tennis—basically things which may stretch the hernia. I do not know if this is accurate information, but it’s how I perceive it to be.”—Umbilical hernia
• “I can not to the more strenuous physical work outdoors that I enjoyed”—Inguinal/femoral hernia
• “It is extremely hard to confidently exercise with a hernia. I can do a handful but would not dream of doing anything too strenuous which rules out activities such as running, jumping, and swimming. My fitness has deteriorated.”—Incisional hernia

### Smoking, Vaping and E-Cigarettes

Fourteen percent of respondents smoked (*n* = 37), and thirteen percent vaped or used e-cigarettes (*n* = 34). Eighty two percent of these respondents (*n* = 58) said that their hernia had no impact on how much they smoked/vaped.

Six respondents recently stopped smoking for health reasons, of which four switched to vaping. A further three smoked or vaped less because of their hernia. Reasons given were pain from coughing (*n* = 3), preparation for surgery (*n* = 1) or other health reasons (*n* = 5).

Six respondents smoked/vaped more (8%) this included two respondents who said that they smoked cannabis for pain relief.

### Alcohol Consumption

Fifty five percent of the respondents said that they consumed alcohol (*n* = 146), of which twenty one percent (*n* = 30) said that they drank less because of their hernia. Two respondents said that they had reduced their alcohol intake to improve their health. Other respondents gave a variety of negative reasons, including pain, reflux, wanting to remain in control, how alcohol caused bloating which could make their hernia “pop out”, and concerns over loose output with a parastomal hernia.

Six respondents said that they drank more alcohol, two because it helped numb their pain, and three because of low mood.

Free text: If affected, please explain why you drink more/less alcohol

• “Alcohol causes bloating which negatively impacts the hernia by causing it to pop out and immense pain”—Incisional hernia
• “Alcohol increases my output. My output is uncomfortable when it’s navigating its way through the twisted bowel in the hernia”—Parastomal hernia
• “Because I want to be in control, no falls, so moderate drinker”—Inguinal/femoral hernia
• “I decided to give up, in the hope it would help in the future”—Incisional hernia
• “Feeling discomfort and not coping with how I look, makes me drink”—Parastomal hernia

### Illegal Drugs

Three percent of respondents (*n* = 7) said they used illegal drugs, of which five stated that they took cannabis (in different forms) for pain relief. Another two respondents said that used cannabis for pain relief, however the drug was legal where they lived.

### Diet

**FIGURE 5:**
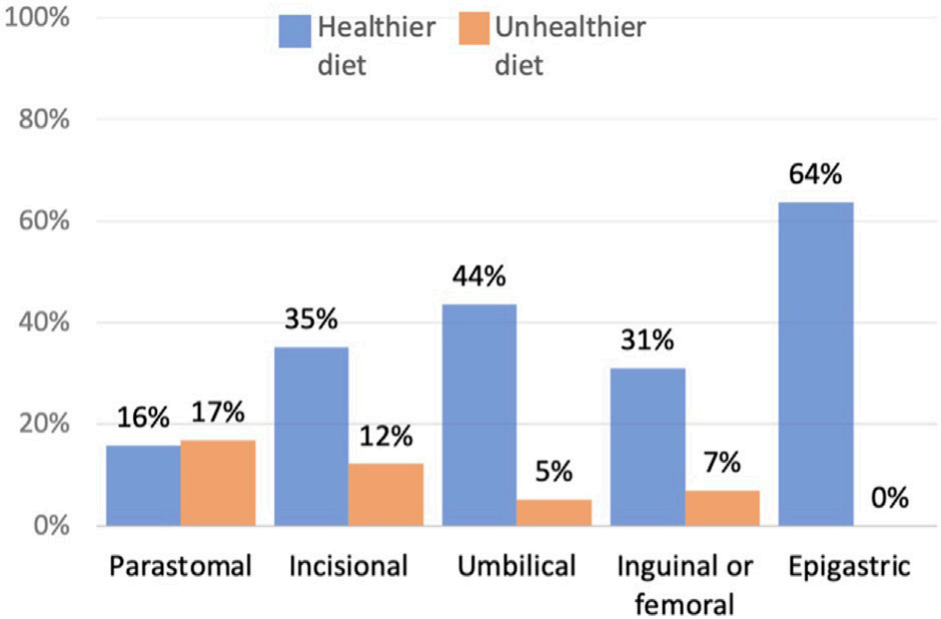
Patients who reported a change in their diet.

Forty-three percent of respondents said that their hernia affected their diet (*n* = 113). The majority of those respondents (71%, *n* = 80) said that their diet was now healthier, the main exception being respondents with parastomal hernias (Figure 5).

**FIGURE 6:**
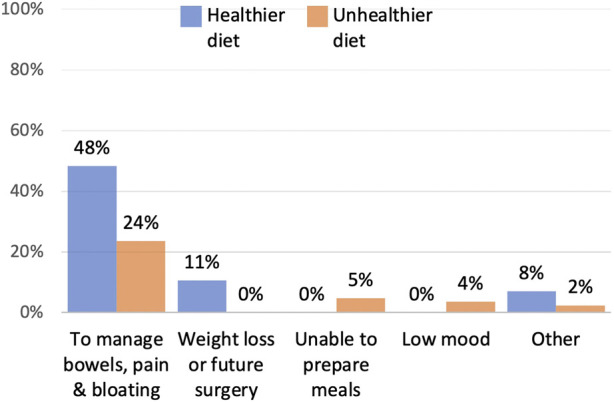
Reasons for change in diet.

Of the respondents who gave information about their diet, only eleven percent (*n* = 9) said that they improved their diet to lose weight or in preparation for surgery. Whereas across all patients who changed their diet (healthier and unhealthier) seventy-two percent (*n* = 61) described how they ate smaller portions or different foods, as an approach to manage their pain, bloating, bowel movements or to reduce the risk of bowel obstructions. Three respondents said that they ate an unhealthy diet due to low mood, four because cooking and shopping was difficult due to pain, and fourteen due to maintaining a low fibre diet to reduce pain or prevent bowel obstructions (Figure 6).

Although respondents described why they changed their diet, most did not clearly document why they considered their diet healthier. Some respondents depicted a balanced diet, however others used phrases such as “easily digestible” which may indicate they are excluding certain fruit, vegetables, and whole grains, or ambiguous phrases such as “clean diet” or “plain diet.” This could indicate that respondents do not know what a healthy diet entails, or it may be that the question was not clearly worded.

Free text: If your hernia affects your diet, please explain how and why

• “I have to eat mostly soft easily digestible or try to keep a clean diet because bloating hurts”—Umbilical hernia—[Reported healthier diet]
• “95% of everything I eat is a careful balance of fibre, protein, and healthy fat. If I can only eat a bit, it is going to be healthy and tasty”—incisional hernia—[Reported healthier diet]
• “All vegetables cause partial blockages, getting past hernia”—Parastomal hernia—[Reported unhealthier diet]
• “Feel uncomfortable if I eat too much”—incisional hernia—[Reported healthier diet]
• “I tried to eat more fruits & fibrous food to ensure smooth BMs without the need for pressure”—Inguinal/femoral hernia—[Reported healthier diet]
• “Find it difficult to walk, shop and stand to cook. Tend to eat quick to prepare convenience food”—incisional hernia—[Reported unhealthier diet]

### Other—Free Text Field

Within the questionnaire there was a free text field, where respondents could discuss any topic that was important to them.

Topics highlighted were

• To raise awareness of the financial burden
• Difficulty sleeping
• How patients’ lives can be on hold whilst waiting for surgery
• The anxiety of a hernia reoccurring
• Whether watchful waiting is “unethical,” if a hernia will only get worse

## Discussion

Only through using validated disease specific QoL tools, which ask questions that are important to patients, can outcomes for different treatment modalities be properly assessed. The literature review by Smith (2) identified six abdominal wall hernia specific QoL tools, each being used to assess different populations, none are universally accepted (1). The measurements covered within these abdominal wall specific QoL tools are summarised by the authors in Table 2. None of these tools cover parastomal hernia specific concerns.

**TABLE 2:** Summary of existing hernia Quality of Life tools.

	AAS	HerQLes	EuraHS-QoL	CCS	HerQL	AHQ
Daily activities inside the house	✓	✓	✓	✓	✓	✓
Pain	—	✓	✓	✓	✓	✓
Physical exercise	✓	✓	✓	✓	✓	—
Appearance	—	—	✓	—	—	✓
Sex	✓	✓	—	—	✓	—
Diet	—	—	—	—	—	—
Social activities	✓	—	—	—	—	—
Employment	✓	✓	—	—	—	—
Economic burden	—	—	—	—	✓	—
Anxiety relating to hernia	—	—	—	—	—	✓
Mental health	—	✓	—	—	—	—

Activities Assessment Scale (AAS) (15).

HerQLes (16).

EuraHS-Qol (17).

Carolinas Comfort Scale (CCS) (18).

HerQL (19).

Abdominal Hernia-Q (AHQ) (20).

This research highlights how existing hernia QoL tools may be limited in the outcomes that they measure. The World Health Organisation (WHO) states that an individual’s QoL incorporates “physical health, psychological state, level of independence, social relationships, personal beliefs, and their relationship to salient features of the environment” (3). Despite this, only one of the existing hernia QoL tools covers mental health, and there is little coverage of an individual’s social wellbeing or the impact their hernia has on sexual relationships (Table 2). Even if generic QoL tools are used in conjunction with hernia specific QoL tools, neither of the commonly used SF-36 (short form 36) and SF-12 (short form 12) surveys cover appearance, sexual intimacy, or diet.

One major function of the abdominal wall is to maintain the anatomical position of the lower digestive organs, it is also involved in the defaecation process (4). Within this study, forty-three percent of patients described how they restricted their diet to manage their bowels, to either reduce pain and bloating, minimise the risk of bowel obstructions or reduce the number of bowel movements. Eating is not just fundamental for life, it goes well beyond physical health. Food is an important part of cultural heritage and national identity, it brings friends and families together (5). Additionally, diet can be used as a tool to manage emotions, like anxiety, stress and depression (6). Interestingly, none of the existing hernia QoL tools directly asked about diet.

Seventy-one percent of respondents who adapted their diet, reported that their diet was now healthier. However, there was insufficient information from those respondents to verify their judgment, and there is the risk that those respondents eating an “easily digestible,” “clean” or “plain” diet are missing key nutrients. Reasons for an unhealthier diet, included being in low mood, reduced mobility and maintaining a low fibre diet. Optimising preoperative nutrition is key to improving recovery after surgery (7). Although further research is needed to understand how a hernia may affect patients’ diet, this study highlights how surgeons may need to assess their patient’s diet and offer support either with healthy eating advice, or through assessment and support from a dietitian.

Although it is recommended not to consume excess alcohol (8), drinking alcohol is a social activity for many, with alcohol playing a role in many social events including parties and celebrations, where psychological benefits include relaxation and increased sociability (9). With twenty three percent of the respondents who consumed alcohol saying that their hernia had a negative impact on their ability to tolerate alcohol consumption, the use of alcohol should be considered when designing QoL tools. This could be asked indirectly within a QoL tool when discussing social activities.

Thirty seven percent (*n* = 98) of respondents said that they had been advised to avoid certain exercise activities, whilst others chose to avoid specific exercises due to fear of making their hernia worse. Further research is needed to understand who is advising those restrictions, whether they are medically correct, and whether with support from surgeons or physiotherapists those restrictions could be lifted. Healthcare professionals could also offer practical advice, such as the use of comfortable support garments, for patients whose exercise is restricted due to the practicalities of their hernia getting in the way.

Several existing QoL tools cover exercise, however they tend to focus on pain and the ability to exercise, maybe this is because exercise is recognised as important for physical health and prehabilitation. However, not being able to exercise may also have repercussions on an individual’s mental health and social wellbeing, particularly if unable to participate in team sports, or group activities like golf or dance which involve significant social interaction.

Sex, intimacy and relationships are an important aspect of social and mental wellbeing (10) but are only covered by three of the hernia QoL tools. Fifty eight percent of respondents to this questionnaire said that their hernia had a negative impact on their sex lives, mainly due to their physical appearance, pain and the practicalities of the hernia getting in the way. Whilst often viewed as a taboo subject, patients waiting for surgery may benefit from practical support such as information on different sex positions, advice on pain management, comfortable support garments or details of flattering underwear. Patients also want to know if their hernia surgery may impact on sexual function (11). Some patients may need counselling, particularly if their hernia was due to trauma, a traumatic event or cancer, as their physical appearance may be a reminder of an event or disease (12). Expectations post-surgery need to be managed, as the patient will continue to have scars and their abdomen may still lack uniformity after the hernia has been repaired, and pain may continue and is reported in up to 23% of patients (13).

The question whether “watchful waiting” is unethical, was raised by one respondent, particularly for large incisional hernias which are known to increase in size over time (14) and may result in a more complex repair. Although a hernia may be classified as asymptomatic from a medical perspective, this research shows how the hernia may impact on the patient’s mental health as well as social and physical relationships. Weight loss is a common reason for delay in hernia repair, however without the appropriate support, the patient may experience a decline in physical health due to reduced physical activity and possibly poor nutritional intake.

## Limitations

This research is a subjective study exploring aspects of QoL deemed important to the patient authors, covering topics which are frequently missed or glossed over in current hernia QoL tools. The results have shown that these aspects are also important to a significant proportion of the surveyed population. However, it is recognised that the respondents to the questionnaire may not be representative, as patients with symptomatic hernias are more likely to be active members within support groups or may be more motivated to respond to help others.

Further limitations lay in the design and distribution of the study. The survey was written by patient authors who have limited experience in research design, and the survey was distributed through social media, which although a useful tool when accessing large populations (11) this may influence the demographics of the respondents.

There is a risk of confirmation bias in the patient quotes selected, the authors have attempted to address this shortcoming through selecting a diverse range of responses covering all hernia types.

## Conclusion

This research has shown how hernias impact on exercise, sex and diet, and how that may have repercussions on mental health as well as social and physical relationships. Without a comprehensive hernia specific QoL tool, which includes these factors, it remains difficult for a surgeon to accurately assess the impact that different treatment modalities may have on patients.

## Data Availability

The raw data supporting the conclusion of this article will be made available by the authors, without undue reservation.
