# Supplementary material for: Quality of Life With a Hernia—A Novel Patient Led Study
Source: J Abdom Wall Surg. 2023 Apr 28;2:11214. doi: 10.3389/jaws.2023.11214 (PMC10831678; doi:10.3389/jaws.2023.11214)
Supplement: Supplementary file 1 [file DataSheet1.PDF]

Living with a hernia

You are being invited to take part in a proposed study. The main aim of this study is to help surgeons understand how hernias impact on patients' wellbeing.

It should take no longer then 5 minutes of your time to complete. The free text fields are optional.

If you no longer have a hernia, please answer the questions based on when you had your hernia.

Some of the questions can be emotionally difficult, including talking about sex, alcohol and substance use

The answers you give will be anonymous. If however you wish to receive the results of this study, then please leave me your email address, which I will only use to send you a summary of the results.

Thank you.

* Required

1. I am *

Mark only one oval.

- Male
- Female
- Other
- Prefer not to say

2. My age is *

Mark only one oval.

- 18-29
- 30-39
- 40-49
- 50-59
- 60-69
- 70 or older
- Prefer not to say

3. I live in *

Mark only one oval.

- UK
- Other European country
- Africa
- Asia
- North America
- South America
- Oceania & Australia
- Prefer not to say

4. Type of hernia? *

Mark only one oval.

- Epigastric (above the belly button)
- Umbilical (belly button)
- Incisional (due to prior surgery)
- Parastomal (under my stoma)
- Inguinal or femoral (in my groin)
- Other

5. Have you had surgery to repair your hernia? *

Mark only one oval.

- No
- Yes - I no longer have a hernia
- Yes, but my hernia reoccured

6. Does having a hernia impact on the amount of alcohol that you drink? *

Mark only one oval.

- Yes, I drink more alcohol
- Yes, I drink less alcohol
- No change
- I don't drink alcohol

7. If affected, please explain why you drink more / less alcohol

8. Does having a hernia affect the amount of cigarettes that you smoke? *

Mark only one oval.

- I don't smoke
- I smoke more because of my hernia
- I smoke less because of my hernia
- No change

9. If your smoking habit has changed because of your hernia, please explain why

10. Does your hernia impact on the amount you vape / e-cigarette? *

Mark only one oval.

- I don't vape
- I vape more because of my hernia
- I vape less because of my hernia
- No change

11. If your vaping / e-cigarette habit has changed because of your hernia, please explain why

12. Do you take any illegal drugs? *

Mark only one oval.

- Yes
- No

13. Please explain if your hernia has any impact on your use of illegal drugs

14. Do you find that you eat more or less because of your hernia? *

Mark only one oval.

- I eat more
- I eat less
- No change

15. If you eat more / less because of your hernia, please explain why

16. Does having a hernia affect the quality of your diet? *

Mark only one oval.

- No
- I eat a healthier diet because of my hernia
- I eat an unhealthier diet because of my hernia

17. If your hernia affects your diet, please explain how and why

18. Does your hernia affect your exercise routine *

Mark only one oval.

- No
- Yes, I exercise more now
- Yes, I am limited in the exercise that I can do

19. If your hernia affects your exercise routine, please explain how and why

20. Have you been told that there are some exercises you should not do, because of your hernia? *

Mark only one oval.

- Yes
- No

21. Please give details of any exercises that you have been told not to do, and why

22. Does your hernia have a negative impact on your sex life (optional question)

Mark only one oval.

- Yes
- No

23. If you feel comfortable discussing this, please explain why your hernia affects your sex life

24. Does your hernia cause you pain, to the extent that you need pain relief? *

Mark only one oval.

- No
- Yes - over the counter medication
- Yes - on prescription
- Yes - I have regular injections
- I use transcutaneous electrical nerve stimulation (TENS)
- Other

25. Is there anything else that you want to tell me?

26. If you would like a summary of the results, please give your email address

Untitled Section

If you you would like to talk to someone regarding alcohol or substance use, further information can be found using the links below
https://www.mind.org.uk/information-support/guides-to-support-and-services/addiction-and-dependency/addiction-and-dependency-resources/
https://www.mhanational.org/conditions/addictionsubstance-use-disorder

This content is neither created nor endorsed by Google.

Google Forms
